# Treatment patterns and humanistic burden of malignant pleural mesothelioma in Spain

**DOI:** 10.1007/s12094-024-03591-5

**Published:** 2024-07-06

**Authors:** Susana Cedres, Julio Calvete, Gavin Taylor-Stokes, Néstor Álvarez Ayerza, David Vilanova Larena, Melinda Daumont

**Affiliations:** 1 Hospital Universitari de La Vall d’Hebron, Barcelona, Spain; 2 Hospital Universitario Puerta del Mar, Cádiz, Spain; 3 Adelphi Real World, Adelphi Mill, Grimshaw Lane, Bollington, Macclesfield, Cheshire UK; 4 Bristol Myers Squibb, Madrid, Spain; 5 Bristol Myers Squibb, Braine L’Alleud, Belgium

**Keywords:** Malignant pleural mesothelioma, Health-related quality of life, Treatment, Spain

## Abstract

**Purpose:**

Malignant pleural mesothelioma (MPM) is an aggressive cancer with long latency and poor prognosis. The real-world treatment patterns and humanistic burden of MPM in an international cohort of patients were recently published. Spanish data are currently lacking and are reported here.

**Methods/Patients:**

Data were collected from three sources: physician-abstracted demographic, clinical and treatment characteristics of patients with MPM; patient-completed questionnaires on treatment satisfaction, symptoms, caregiver use, and impact of the disease; and caregiver-completed questionnaire reporting their activity and its impact on their daily life.

**Results:**

The 241 patients in Spain were primarily elderly (median age: 67 years), male, retired/unemployed/on long-term sick leave, and diagnosed at stage IV with unresectable disease. Exposure to asbestos was detected (54%, 101/188). First-line treatment (1L) consisted primarily of doublet chemotherapy (86%, 207/241). Of 102 patients who completed 1L at data abstraction, 67 were receiving maintenance therapy, most commonly singlet chemotherapy with pemetrexed. Best supportive care was given to 29 patients, primarily after 1L (86.2%, 25/29). Symptom burden was high and health-related quality of life was poor and declined with progression: mean (SD) EQ-5D score and EQ-5D visual analogue scale score were 0.615 (0.285) and 60.8 (17.1) in 1L and 0.497 (0.370) and 56.1 (19.5) in second line. Overall, 67% of patients (162/241) required daily assistance from their caregiver, who reported an impact on their psychological well-being.

**Conclusions:**

Patients with MPM in Spain were overall treated according to treatment guidelines at the time. Nevertheless, a considerable burden of disease was reported by patients and caregivers.

**Supplementary Information:**

The online version contains supplementary material available at 10.1007/s12094-024-03591-5.

## Introduction

Malignant pleural mesothelioma (MPM) is an aggressive and rare cancer that arises from the mesothelial cells that line the pleura [1, 2]. MPM predominantly occurs in men and is diagnosed at a median age of 63 years [3]. MPM has a latency of several decades and is strongly associated with prior occupational exposure to asbestos [1, 2]. The delayed appearance and often challenging diagnosis [1] result in MPM generally presenting at advanced stage, when prognosis is dismal [2, 4].

The incidence of MPM varies widely across world regions and is associated with higher development index, gross domestic product, and asbestos exposure [5]. The worldwide age-standardised rate of MPM was 0.30 per 100,000 persons in 2020; Europe, Australia and New Zealand, and South Africa had the highest incidence [5]. In Spain, age-standardised incidence of MPM was 0.53 per 100,000 persons in 2020 [6].

MPM can be categorised in three subtypes according to histology; the epithelioid subtype is the most common (60–70%), followed by sarcomatoid and biphasic [1, 2]. Histology has treatment implications, for example excluding patients from surgical intervention if there is a sarcomatoid component in the tumour [1]. Histology subtypes have also been associated with prognosis, with epithelioid tumours having better overall and progression-free survival [7, 8].

Over the past two decades, little progress had been made in finding new treatment strategies; the main therapeutic option consisted of combinations with platinum-based chemotherapy, associated with median overall survival of 11–12 months [2, 9, 10]. Recently, the CheckMate 743 study showed immunotherapy with nivolumab plus ipilimumab led to an advantage in overall survival in patients with MPM (~ 18 months), regardless of histology, compared to chemotherapy [11], and this combination is now approved for MPM by the European Medicines Agency and the U.S. Food and Drug Administration [12, 13]. The phase 3 studies CheckMate 743 and IND227 also found that the overall survival benefit of chemotherapy was greater for epithelioid histology than non-epithelioid histology [11, 14]. Moreover, there are currently no predictive biomarkers to identify patients who will benefit from treatment [15].

Patients with MPM have a considerable symptom burden, including pain, weakness, dyspnoea and poor well-being [16], which negatively impact quality of life [17]. MPM also affects the quality of life of caregivers, who experience physical and psychological problems that worsen with an increasingly longer caregiving period [17–19]. Caregiving also affects employment by deteriorating working conditions [18, 19].

In light of the geographic and clinical variability of MPM and its impact on patients’ quality of life, we recently evaluated the treatment patterns and humanistic burden of MPM on patients and caregivers in an international cohort [20]. Here, we present the results of the Spanish cohort.

## Methods

### Study design and population

Here, we evaluated the data of patients with MPM in Spain, which is a subset of a larger cohort from an international study that also included patients from France, Germany, Italy, and the United Kingdom. The study design (Supplementary Fig. 1) and methodology have been previously described [20]. Briefly, data were collected between January and June 2019 using electronic case report forms (eCRF) completed by the treating physicians, voluntary patient self-completion questionnaires (PSC), and voluntary caregiver self-completion questionnaires (CSC). Participating physicians had been practising for ≥ 5 and ≤ 35 years. Patients were ≥ 18 years old, had a confirmed diagnosis of unresectable MPM, and were undergoing or had completed first-line (1L) treatment with anti-cancer systemic therapy. Patients enrolled in a clinical trial were excluded. The study was approved by the Western Institutional Review Board (IRB Number: 20183141).

### Outcomes

Physicians abstracted patient information from the medical records into eCRF at the time of consultation. The eCRF included demographic and clinical characteristics, treatment history, adverse events of current regimen, visits, and hospitalisations. The PSC also collected information on treatment and adverse events, as well as treatment satisfaction, health status, caregiver use, and impact of the disease on caregiving needs, and time and financial burden. Three patient-reported outcome measures were included in the PSC: (1) EQ-5D-3L, which measures general health-related quality of life (HRQoL) using a descriptive system with five dimensions (mobility, self-care, usual activities, pain/discomfort, and anxiety/depression), where the index score ranges from negative values (“worse than death health state) to 1 (perfect health), and a visual analogue scale (VAS), where scores range from 0 (worst imaginable health) to 100 (best imaginable health); (2) LCSS-Meso (Lung Cancer Symptom Scale-Mesothelioma), a mesothelioma-specific instrument to evaluate HRQoL and symptom burden where scores range from 0 (better quality of life) to 100 (worse quality of life), except for LCSS 3-IGI (3-item global index), where scores range from 0 to 300, with higher scores indicating better quality of life; and (3) WPAI (Work Productivity and Activity Impairment) questionnaire, a six-item instrument to evaluate the impact of the disease on work productivity and usual activities, represented as a percentage. The CSC collected demographic characteristics, caregiver use, impact of caregiving, and time and financial burden of caregiving. The CSC also included the WPAI questionnaire and ZBI (Zarit Burden Interview), a 22-item questionnaire to evaluate caregivers’ perception of the burden of the disease, with scores ranging from 0 to 88 (0–21, no to mild burden; 21–40; mild to moderate burden; 41–60, moderate to severe burden; ≥ 61, severe burden).

### Data analysis

Data were analysed descriptively, as previously reported [20], using means and standard deviations (SD) for continuous numerical variables, and counts and proportions for categorical variables. Outcomes were stratified by patients’ current line of treatment (1L, maintenance therapy, second line [2L], or best supportive care after therapy) or tumour histology (epithelioid vs non-epithelioid; the latter combined data for biphasic and sarcomatoid histology, as well as unknown histology). The minimal important difference (MID) is the smallest clinically meaningful change in a parameter. MID for 0.08 points for EQ-5D-3L, 7 points for EQ-5D VAS, 10 points for LCSS individual items and average symptom burden index, and 30 points for LCSS 3-IGI.

## Results

### Study population

Thirty physicians, all oncologists, participated in Spain; 53% (16/30) were female, and 43% (13/30) had never been involved in a MPM clinical trial. The physicians practiced primarily in a public hospital (75% of the time of the overall sample was devoted to this setting), followed by a comprehensive cancer centre (17.3% of the time); they practiced in a private hospital only 0.3% of their time. Physicians abstracted data from 241 patients in Spain, of which 87% (209/241) completed PSCs and 46% (111/241) had CSCs. Overall, patients in Spain had a mean (range) age of 65.7 (40–90) years; 72% (174/241) of patients were male, 63% (153/241) were underweight or had healthy weight (body mass index [BMI] < 25), and 73% (177/241) were current or former smokers (Table 1). Most patients presented the epithelioid histologic subtype (64% [155/241]); 17% (40/241) of patients had the biphasic subtype, 13% (31/241) the sarcomatoid subtype, and subtype was unknown for 6% (15/241) of patients. Most patients, 83% (199/241), were retired, unemployed, or on long-term sick leave, which were caused by MPM in over a quarter of these cases (28%, 56/199). Overall, 75% (180/241) of patients had ECOG PS 0–1; most patients (69%, 167/241) were diagnosed with stage IV MPM and largely had unresectable disease (88%, 213/241). Hypertension was the most frequent comorbidity (42%, 102/241). Exposure to asbestos was frequent among patients with MPM (54%, 101/188) and few had been exposed to erionite (2%, 3/128), although information on the latter was missing for almost half of the sample. Patients were seen by a doctor a mean (SD) 1.9 (1.5) months after they first experienced symptoms, and they received a diagnosis 1.7 (2.9) months after the first visit to the doctor. In general, patient characteristics were evenly distributed between the epithelioid and non-epithelioid subtypes.

**Table 1:** Patient demographic and clinical characteristics

Parameter	Overall (N = 241) n (%)	Epithelioid histology (N = 155) n (%)	Non-epithelioid histology (N = 86) n (%)
**Age (years); median (range)**	67.0 (40.0–90.0)	67.0 (40.0–90.0)	66.3 (40.0–90.0)
**Age category**			
< 70 years	151 (63)	99 (64)	52 (60)
≥ 70 years	90 (37)	56 (36)	34 (40)
**Sex**			
Male	174 (72)	115 (74)	59 (69)
Female	67 (28)	40 (26)	27 (31)
**BMI**			
< 25	153 (63)	93 (60)	60 (70)
≥ 25	88 (37)	62 (40)	26 (30)
**Smoking status**			
Current smoker	27 (11)	15 (10)	12 (14)
Former smoker	150 (62)	101 (65)	49 (57)
Never smoker	64 (27)	39 (25)	25 (29)
**History of asbestos exposure**			
Yes	101 (42)	61 (39)	40 (47)
No	87 (36)	58 (37)	29 (34)
Unknown/missing	53 (22)	36 (23)	17 (20)
**History of erionite exposure**			
Yes	3 (1)	1 (1)	2 (5)
No	125 (52)	84 (54)	41 (48)
Unknown/missing	113 (47)	70 (45)	43 (50)
**MPM stage at diagnosis**			
Stage 1	5 (2)	4 (3)	1 (1)
Stage 2	14 (6)	8 (5)	6 (7)
Stage 3	54 (22)	35 (23)	19 (22)
Stage 4	167 (69)	108 (70)	59 (69)
Unable to stage	1 (0)	0 (0)	1 (1)
**ECOG at initial diagnosis**			
0	36 (15)	23 (15)	13 (15)
1	144 (60)	97 (63)	47 (55)
2	56 (23)	34 (22)	22 (26)
3	4 (2)	1 (1)	3 (3)
4	0 (0)	0 (0)	0 (0)
Unknown/missing	1 (0)	0 (0)	1 (0)
**ECOG at diagnosis of unresectable MPM**	N = 74	N = 47	N = 27
0	5 (7)	2 (4)	3 (11)
1	46 (62)	30 (64)	16 (59)
2	22 (30)	15 (32)	7 (26)
Unknown/missing	1 (0)	0 (0)	1 (0)
**Resection status at diagnosis**	N = 241	N = 155	N = 86
Resectable	19 (8)	15 (10)	4 (5)
Unresectable	213 (88)	136 (88)	77 (90)
Unknown/missing	9 (4)	4 (3)	5 (6)
**Comorbidities (≥ 10% of patients)**			
Peripheral vascular disease	24 (13)	13 (8)	11 (13)
Hypertension	102 (42)	65 (42)	37 (43)
Chronic pulmonary disease	30 (12)	20 (13)	10 (12)
Diabetes	47 (20)	27 (17)	20 (23)
COPD	62 (26)	42 (27)	20 (23)
**Employment status**			
Working (part time or full time)	18 (7)	9 (6)	9 (10)
Homemaker	23 (10)	13 (8)	10 (12)
On long-term sick leave, retired, or unemployed	199 (83)	133 (86)	66 (77)
On long-term sick leave, retired, or unemployed because of MPM	56 (28)	38 (29)	18 (21)
Unknown/missing	1 (0)	0 (0)	0 (0)

Percentages may not add to 100% because of rounding

*COPD* chronic obstructive pulmonary disease, *MPM* malignant pleural mesothelioma

The demographic and clinicopathological characteristics of patients with MPM in Spain were similar to those of patients abroad (Germany, France, Italy and the UK) in terms of age, sex, BMI and MPM stage at diagnosis (Supplementary Fig. 2). The Spanish sample had fewer patients that were currently smokers and had more patients with resectable disease at diagnosis. Considering only available data, 54% of patients in Spain had been exposed to asbestos vs 79% abroad, and 2% of patients in Spain had been exposed to erionite vs 6% abroad.

Tumour biomarker information was available for 29% (71/241) of patients overall (28%, 44/155 with epithelioid histology; 31%, 27/86 with non-epithelioid histology); in these, the most common biomarker detected (positive expression and/or mutations) was BAP1 (50%, 2/4), followed by PD-L1 (29%, 18/63), VEGF (7%, 1/14), ALK (5%, 2/44) and EGFR (2%, 1/51) (Supplementary Table 1).

### Treatment patterns

At 1L, 86% (207/241) of patients were treated with doublet chemotherapy, most commonly cisplatin plus pemetrexed (56%, 134/241), followed by carboplatin plus pemetrexed (30%, 73/241) (Table 2). Patients who completed 1L (*n* = 116) received primarily doublet chemotherapy (88%, 102/116), with a mean (SD) 5.2 (1.4) cycles. The most common reasons cited by physicians for selecting a specific 1L treatment regimen were progression-free survival benefit (66%, 158/241), manageable toxicity profile (57%, 137/241), and familiarity with treatment (52%, 125/241) (Supplementary Fig. 3).

**Table 2:** Treatment patterns

Parameter	Sample size n (%)
**1L treatment**	N = 241
Doublet chemotherapy	207 (86)
Carboplatin plus pemetrexed	73 (30)
Cisplatin plus pemetrexed	134 (56)
Bevacizumab plus chemotherapy	5 (2)
Single-agent chemotherapy	23 (10)
Other	6 (2)
Immunotherapy	1 (0)
Other chemotherapy combinations	3 (1)
Pemetrexed plus other chemotherapy	1 (0)
**1L maintenance treatment**	N = 67
Pemetrexed	60 (90)
Other	7 (10)
**Best supportive care after 1L**	N = 25
Opioids	18 (72)
Analgesics other than opioids	14 (56)
Pleural aspiration/drainage	8 (32)
Non-pharmacological interventions	7 (28)
Radiotherapy	5 (20)
Denosumab	3 (12)
Bisphosphonates	1 (4)
Watch and wait approach	1 (4)
**2L treatment after systemic anti-cancer therapy**	N = 39
Single-agent chemotherapy	26 (67)
Gemcitabine	9 (23)
Vinorelbine	12 (31)
Other	5 (3)
Doublet chemotherapy	1 (3)
Other	12 (31)
Immunotherapy	2 (5)
Other chemotherapy combinations	9 (23)
Raltitrexed plus chemotherapy	1 (3)

At the time of data abstraction, 66% (67/102) of the patients who completed 1L had received/were receiving maintenance therapy, most commonly singlet chemotherapy (93%, 62/67), mainly pemetrexed (90%, 60/67). Systemic anti-cancer therapy was given to 39 patients who initiated second-line treatment; 67% (26/39) of patients received singlet chemotherapy, most commonly vinorelbine (31%, 12/39) and gemcitabine (23%, 9/39) (Table 2). Best supportive care was given to 29 patients, primarily after 1L (86%, 25/29), with opioids being the most common treatment (72%, 18/25) (Table 2).

In total, 13% (31/241) of patients had undergone thoracic surgery prior to their treatment for MPM; 9% (22/241) and 10% (25/241) of patients had ever received radiotherapy or surgery, respectively, for MPM (including palliative and adjunctive therapy). All patients with diagnosis of resectable disease for whom data were available (n = 19) underwent surgery. Radical pleurectomy was the most common type of surgery (61%, 14/23), followed by resection (22%, 5/23), pleurectomy (9%, 2/23), and pneumonectomy (4%, 1/23); 4% (1/23) of patients had other type of surgery. The main reason for surgery was primary treatment in 78% (18/23) of patients; surgery was used in addition to chemotherapy in 4% (1/23) of patients and for other reasons in the remaining 17% (4/23) of patients.

The treatment guidelines most commonly used by physicians to make treatment decisions were those developed by ESMO (83%, 25/30), followed by NCCN (73%, 22/30), ASCO (70%, 21/30), and national Spanish guidelines (40%, 12/30).

### Burden of the disease

**Fig. 1:**
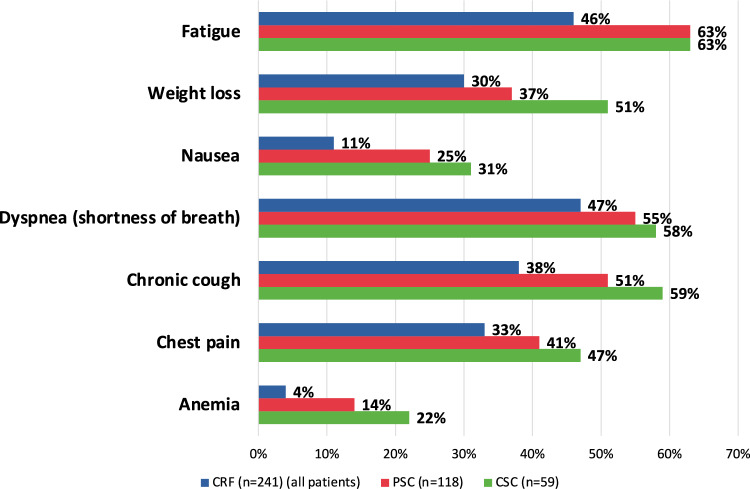
Symptoms experienced by > 10% patients receiving first-line treatment. *CRF* case report form, *CSC* caregiver self-completion questionnaire, *PSC* patient self-completion questionnaire

The impact of MPM on patients’ daily activities was evidenced by 76% (158/209) reporting an impact sometimes, most of the time or always, which was similar across treatment groups (doublet, triplet, singlet, best supportive care) (Supplementary Fig. 4). Symptoms experienced by patients receiving 1L treatment were consistent across the three data sources; however, in all cases except for fatigue, caregivers reported a higher rate of symptoms than patients, who reported a higher rate of symptoms than were collected in the eCRF. The most common symptoms were fatigue, chronic cough, dyspnoea, weight loss, chest pain, nausea, and anaemia (Fig. 1). There were no grade 4 symptoms. Overall, only 3–4% of patients were asymptomatic. At the time of data abstraction, 35% (85/241) of patients were experiencing side effects of their current treatment (in any line), as collected in their eCRF. The most common side effects were anaemia (68%, 58/241), fatigue (59%, 30/241) and nausea (53%, 45/241) (Supplementary Table 2).

Thirty-eight patients had been hospitalised since initiating their current treatment for MPM. Mean (SD) duration of a hospital stay was 8.7 (6.8) days, mainly due to an emergency for 61% (23/38) of patients; other hospitalisations were elective for 29% (11/38) of patients or a standard procedure for 16% (6/38) of patients. Since initiating their current treatment for MPM, patients visited multiple specialists and healthcare professionals, most frequently medical oncologists for a mean (SD) of 7.5 (6.3) days, pulmonologists for 1.4 (1.3) days, primary care physicians for 1.7 (3.0) days, palliative care physicians for 0.4 (1.5) days, and cancer nurse specialists for 2.2 (4.5) days. Procedures conducted since initiating the patients’ current treatment included a mean (SD) of 2.0 (1.9) chest X-rays and 2.2 (1.7) computerised tomography scans.

### Health-related quality of life

**Fig. 2:**
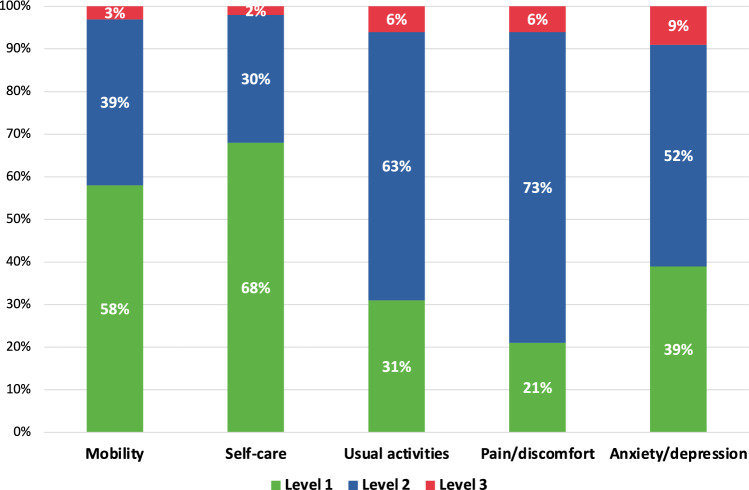
EQ-5D profile of patients receiving first-line treatment. Mobility: Level 1, I have no problems in walking about; Level 2, I have some problems in walking about; Level 3, I am confided to bed. Self-care: Level 1, I have no problems with self-care; Level 2, I have some problems washing or dressing myself; Level 3, I am unable to wash or dress myself. Usual activities: Level 1, I have no problems with performing my usual activities; Level 2, I have some problems with performing my usual activities; Level 3, I am unable to perform my usual activities. Pain/discomfort: Level 1, I have no pain or discomfort; Level 2, I have moderate pain or discomfort; Level 3, I have extreme pain or discomfort. Anxiety/depression: Level 1, I am not anxious or depressed; Level 2, I am moderately anxious or depressed; Level 3, I am extremely anxious or depressed

Data on HRQoL per EQ-5D-3L available for patients currently at 1L (n = 118) or 2L (n = 56) showed an overall mean (SD) EQ-5D-3L score of 0.615 (0.285) and 0.497 (0.370), respectively, and EQ-5D VAS mean (SD) score of 60.8 (17.1) and 56.1 (19.5), respectively. The difference in EQ-5D-3L score was clinically meaningful between 1 and 2L, and both EQ-5D-3L and EQ-5D VAS had clinically meaningful differences compared with population norms for Spain [21]. The EQ-5D health profile of patients undergoing first-line treatment revealed the greatest level of impairment was experienced in pain/discomfort and usual activities (Fig. 2). Data on LCSS were collected for 209 patients across 1L, 1L maintenance, and 2L. LCSS mean (SD) score was 45.8 (17.8) in 1L and 50.5 (21.2) in 2L (Supplementary Fig. 5). LCSS 3-IGI scores were 159.1 in 1L and 141.9 in 2L. All LCSS scores showed a worse quality of life for patients in 2L than in 1L, with no clinically meaningful difference. The WPAI questionnaire revealed a mean (SD) 52.5% (24.6) degree of impairment (n = 197).

Regarding the time burden of the disease, patients reported their physician consultations lasted a mean (SD) 46.5 (60.3) minutes. Patients travelled a mean (SD) 12.7 (14.7) km to receive treatment. Moreover, 43% (89/209) of patients reported paying for prescription medicine for MPM or other conditions.

### Caregiver profile and burden of the disease

According to physicians, 67% of patients (162/241) required daily assistance from their caregiver. Caregivers had a mean (SD) age of 58.0 (12.9) years, were primarily women (78%, 87/111), generally lived with the patient (77%, 86/111), were most commonly a partner/spouse (63%, 70/111), and had variable employment status (Table 3).

**Table 3:** Caregiver demographics

Parameter	N = 111 n (%)
**Age (years); mean [SD]**	58.0 [12.9]
**Age category**	
< 65 years	63 (57)
≥ 65 years	48 (43)
**Sex**	
Male	22 (20)
Female	87 (78)
Unknown/missing	2 (2)
**Caregiver lives with patient**	
Yes	86 (77)
No	24 (22)
Unknown/missing	1 (1)
**Relationship with patient**	
Partner/spouse	70 (63)
Child	16 (14)
Sibling	10 (9)
Other	15 (14)
**Employment status**	
Work full time	31 (28)
Work part time	12 (11)
Student	
Homemaker	24 (22)
Retired	35 (32)
Unemployed	5 (5)
Other	4 (4)

Sum of percentages may not equal 100% because of rounding

Data reported by caregivers showed approximately half (55%, 61/111) required treatment for conditions that were developed or exacerbated by their caregiver role, most frequently sleeping problems (20%, 12/61), anxiety (15%, 9/61), stress (13%, 8/61), and depression (11%, 7/61). Caregivers missed a mean (SD) 43.1 (78.1) hours of work in the most recent month because of their caregiver role. In line with this, 26% (29/111) of caregivers reported a negative impact on their financial situation or that of the family. Employed caregivers reported the following main lifestyle changes since becoming a caregiver: decreased social activities (51%, 22/43), reduced time for themselves (60%, 26/43), reduced time they give to other family members (35%, 15/43), and decreased level of fitness (26%, 11/43)–19% (8/43) of caregivers reported no lifestyle changes.

Caregivers devoted a mean (SD) 9.2 (7.5) daily hours of emotional and/or physical support to the patient. Most common activities that caregivers assisted patients with included emotional support/encouragement (83%, 92/111), reminding to take medication (61%, 68/111), and remind or make appointments (52%, 59/111) (Table 4). Providing emotional support/encouragement was regarded by many caregivers (65%, 72/111) as the most time-intensive activity. The mean (SD) degree of impairment of overall caregiver activity because of the patient’s condition was 40.3% (23.9). The mean (SD) ZBI score was 34.8 (15.5), over the threshold of 24 that indicates risk of developing depression.

**Table 4:** Activities caregivers assist patients with

Parameter	N = 111 n (%)
**Daily activities**	
Emotional support/encouragement	92 (83)
Remind patient to take medication	68 (61)
Travelling out of home	62 (56)
Remembering/making appointments	59 (53)
Help with preparing meals/cooking food	58 (52)
Drive patient to work/hospital/appointment	55 (50)
Help with shopping	47 (42)
Help advising on treatment options	37 (33)
Help giving treatment	34 (31)
Getting dressed/washed	32 (29)
Help plan and organise everyday activities	28 (25)
Help patient research their condition	27 (24)
Help with walking short distances	25 (23)
Look after children	25 (23)
Communicate with others	24 (22)
Help with going to the toilet	20 (18)
Finances/money	17 (15)
Using household appliances	15 (14)
Assist with eating/feeding the patient	16 (14)
Help getting in and out of bed	14 (13)
Assist with moving the patient's oxygen tank	13 (12)
Help with reading (e.g., books, newspapers)	7 (6)
**Most time-intensive activities**	
Emotional support/encouragement	72 (65)
Help advising on treatment options	21 (19)
Remind patient to take medication	19 (17)
Drive patient to work/hospital/appointment	20 (18)
Getting dressed/washed	18 (16)
Travelling out of home	18 (16)
Remembering/making appointments	12 (11)

## Discussion

In this study we found that most patients with MPM in Spain were elderly and male and had generally been diagnosed with MPM at an advanced and unresectable stage. Patients reported a considerable degree of impairment as a result of the disease, with worse HRQoL scores reported in later lines of treatment. The epithelioid histologic subtype was the most common in this sample, as expected [1], and there were no marked differences in demographic or clinical characteristics between patients with epithelioid or non-epithelioid histology. Most patients received treatment according to clinical guidelines for 1L at the time of the study, i.e., platinum-based doublet chemotherapy. Singlet chemotherapy was the most common maintenance therapy after 1L and it was also frequently used in 2L, where other options such as immunotherapy and chemotherapy combinations were used. These findings are in line with those of a larger cohort of patients with MPM in Spain [22] and also with the recently published findings of the international cohort study [20]. Tumour biomarker information was available for less than a third of the sample, with BAP1 and PD-L1 being the most common. Further research is needed to elucidate the role of tumour biomarkers in disease progression.

At the time of data abstraction, chemotherapy was the standard of care treatment for MPM in clinical guidelines, and the treatment patterns identified in this study reflect this pattern. Since then, immunotherapy has also become a recommended 1L treatment [15]. The treatment landscape for patients with MPM will continue to change in the coming years, as recent studies have shown that 1L treatment with immunotherapy alone [11] or combined with chemotherapy [14, 23] improved outcomes for patients with MPM compared with chemotherapy [14, 23] Immunotherapy is also a valuable option for 2L after progression on chemotherapy [24]. Other treatments, such as bevacizumab plus cisplatin and pemetrexed, have also demonstrated improved outcomes [25], although this combination is currently not approved.

The findings of the Spanish cohort are, overall, similar to those of the international cohort, with some exceptions. In the Spanish cohort, 54% of patients had been exposed to asbestos, similar to that reported in another study in Spain (45%) [22]. In both cases, the rates in Spain were lower than those found abroad (79%) and in the overall international cohort of the study described here (75%) [20]. Moreover, maintenance therapy after 1L was given to 66% (67/102) of patients who completed 1L in Spain and to 51% of patients in the international cohort [20]. In both cases, pemetrexed was the most common treatment used. Maintenance therapy, thus, appears to be a common approach followed for patients with MPM, despite the lack of approval of agents for this use.

We found a considerable symptom burden and effect on HRQoL in patients with MPM in Spain. Additionally, patients identified more than one caregiver, suggesting an even higher burden. Caregivers also reported a burden of the disease in terms of psychological and physical aspects, as well as an impact on employment. Caregivers were primarily women who were the patient’s partner/spouse—adult children were also caregivers to a lesser extent—and lived with the patient. These findings are in line with those from the international cohort [19] and with other reports of quality of life of caregivers of patients with cancer [18].

This study’s strengths and limitations have been previously published with the findings of the international cohort [20]. Briefly, the main strength of this study was the inclusion of a large real-world cohort of patients, considering the rarity of the disease. Additionally, data were obtained from multiple sites. The main limitations of this study were the cross-sectional design and the reliance on patient- or caregiver-reported data, as well as on complete physician-collected information. Another limitation is the relatively short timeframe (6 months) in which the study was conducted.

In conclusion, MPM remains a disease with poor prognosis and reduced HRQoL, which impacts both patients and caregivers. Multidimensional management of MPM is of interest, considering the rarity of the disease and its burden. Most patients develop chemotherapy resistance and have short responses, and maintenance therapy is not approved. New strategies such as immunotherapy have shown promising results and should be considered.

## Supplementary Information

Below is the link to the electronic supplementary material.

Supplementary file1 (DOCX 361 KB)

Supplementary file2 (DOCX 26 KB)

## Data Availability

Not applicable.

## References

[1] Fels Elliott DR, Jones KD. Diagnosis of mesothelioma. Surg Pathol Clin. 2020;13:73–89. 32005436 10.1016/j.path.2019.10.001

[2] Janes SM, Alrifai D, Fennell DA. Perspectives on the treatment of malignant pleural mesothelioma. N Engl J Med. 2021;385:1207–18. 34551230 10.1056/NEJMra1912719

[3] Rusch VW, Giroux D, Kennedy C, Ruffini E, Cangir AK, Rice D, et al. Initial analysis of the international association for the study of lung cancer mesothelioma database. J Thorac Oncol. 2012;7:1631–9. 23070243 10.1097/JTO.0b013e31826915f1

[4] National Cancer Institute. SEER*Explorer: An interactive website for SEER cancer statistics. 2023. https://seer.cancer.gov/statistics-network/explorer/application.html. Accessed 8 Oct 2023.

[5] Huang J, Chan SC, Pang WS, Chow SH, Lok V, Zhang L, et al. Global incidence, risk factors, and temporal trends of mesothelioma: a population-based study. J Thorac Oncol. 2023;18:792–802. 36775192 10.1016/j.jtho.2023.01.095

[6] Ferlay J, Ervik M, Lam F, Colombet M, Mery L, Piñeros M, et al. Global cancer observatory: cancer today. Lyon, France: International Agency for Research on Cancer. In: Global Cancer Observatory. 2020. https://gco.iarc.fr/today. Accessed 8 Oct 2023.

[7] Meyerhoff RR, Yang CFJ, Speicher PJ, Gulack BC, Hartwig MG, D’Amico TA, et al. Impact of mesothelioma histologic subtype on outcomes in the surveillance, epidemiology, and end results database. J Surg Res. 2015;196:23–32. 25791825 10.1016/j.jss.2015.01.043 PMC4430361

[8] Cedres S, Assaf JD, Iranzo P, Callejo A, Pardo N, Navarro A, et al. Efficacy of chemotherapy for malignant pleural mesothelioma according to histology in a real-world cohort. Sci Rep. 2021;11:21357. 34725384 10.1038/s41598-021-00831-4 PMC8560806

[9] Vogelzang NJ, Rusthoven JJ, Symanowski J, Denham C, Kaukel E, Ruffie P, et al. Phase III study of pemetrexed in combination with cisplatin versus cisplatin alone in patients with malignant pleural mesothelioma. J Clin Oncol. 2003;21:2636–44. 12860938 10.1200/JCO.2003.11.136

[10] Van Meerbeeck JP, Gaafar R, Manegold C, Van Klaveren RJ, Van Marck EA, Vincent M, et al. Randomized phase III study of cisplatin with or without raltitrexed in patients with malignant pleural mesothelioma: an intergroup study of the European organisation for research and treatment of cancer lung cancer group and the National Cancer Institute. J Clin Oncol. 2005;23:6881–9. 16192580 10.1200/JCO.20005.14.589

[11] Baas P, Scherpereel A, Nowak AK, Fujimoto N, Peters S, Tsao AS, et al. First-line nivolumab plus ipilimumab in unresectable malignant pleural mesothelioma (CheckMate 743): a multicentre, randomised, open-label, phase 3 trial. Lancet. 2021;397:375–86. 33485464 10.1016/S0140-6736(20)32714-8

[12] OPDIVO® (nivolumab) [package insert]. Princeton NJ:Bristol-Myers Squibb Company; 2023.

[13] OPDIVO® (nivolumab). European Medicines Agency SmPC. Dublin, Ireland:Bristol-Myers Squibb Pharma EEIG; 2023.

[14] Chu Q, Perrone F, Greillier L, Tu W, Piccirillo MC, Grosso F, et al. Pembrolizumab plus chemotherapy versus chemotherapy in untreated advanced pleural mesothelioma in Canada, Italy, and France: a phase 3, open-label, randomised controlled trial. Lancet. 2023;402:2295–306. 37931632 10.1016/S0140-6736(23)01613-6

[15] Popat S, Baas P, Faivre-Finn C, Girard N, Nicholson AG, Nowak AK, et al. Malignant pleural mesothelioma: ESMO clinical practice guidelines for diagnosis, treatment and follow-up☆. Ann Oncol. 2022;33:129–42. 34861373 10.1016/j.annonc.2021.11.005

[16] Mercadante S, Degiovanni D, Casuccio A. Symptom burden in mesothelioma patients admitted to home palliative care. Curr Med Res Opin. 2016;32:1985–8. 27532369 10.1080/03007995.2016.1226165

[17] Granieri A, Tamburello S, Tamburello A, Casale S, Cont C, Guglielmucci F, et al. Quality of life and personality traits in patients with malignant pleural mesothelioma and their first-degree caregivers. Neuropsychiatr Dis Treat. 2013;9:1193–202. 23983468 10.2147/NDT.S48965 PMC3748052

[18] Guerra-Martín MD, Casado-Espinosa MDR, Gavira-López Y, Holgado-Castro C, López-Latorre I, Borrallo-Riego Á. Quality of life in caregivers of cancer patients: a literature review. Int J Environ Res Public Health. 2023;20:1570. 36674325 10.3390/ijerph20021570 PMC9863368

[19] Moore A, Bennett B, Taylor-Stokes G, Daumont MJ. Caregivers of patients with malignant pleural mesothelioma: who provides care, what care do they provide and what burden do they experience? Qual Life Res. 2023;32:2587–99. 37097405 10.1007/s11136-023-03410-4 PMC10393857

[20] Moore A, Bennett B, Taylor-Stokes G, McDonald L, Daumont MJ. Malignant pleural mesothelioma: treatment patterns and humanistic burden of disease in Europe. BMC Cancer. 2022;22:693. 35739480 10.1186/s12885-022-09750-7 PMC9229520

[21] Szende A, Janssen B, Cabasés J. Self-reported population health: an international perspective based on EQ-5D. Springer Open. 2014. 29787044

[22] Remon J, Nadal E, Dómine M, Ruffinelli J, García Y, Pardo JC, et al. Malignant pleural mesothelioma: treatment patterns and outcomes from the Spanish Lung Cancer Group. Lung Cancer. 2020;147:83–90. 32682189 10.1016/j.lungcan.2020.06.034

[23] Chu QS, Piccirillo MC, Greillier L, Grosso F, Lo Russo G, Florescu M, et al. IND227 phase III (P3) study of cisplatin/pemetrexed (CP) with or without pembrolizumab (pembro) in patients (pts) with malignant pleural mesothelioma (PM): a CCTG, NCIN, and IFCT trial. J Clin Oncol. 2023;41:LBA8505.

[24] Fennell DA, Ewings S, Ottensmeier C, Califano R, Hanna GG, Hill K, et al. Nivolumab versus placebo in patients with relapsed malignant mesothelioma (CONFIRM): a multicentre, double-blind, randomised, phase 3 trial. Lancet Oncol. 2021;22:1530–40. 34656227 10.1016/S1470-2045(21)00471-X PMC8560642

[25] Zalcman G, Mazieres J, Margery J, Greillier L, Audigier-Valette C, Moro-Sibilot D, et al. Bevacizumab for newly diagnosed pleural mesothelioma in the mesothelioma Avastin Cisplatin Pemetrexed Study (MAPS): a randomised, controlled, open-label, phase 3 trial. Lancet. 2016;387:1405–14. 26719230 10.1016/S0140-6736(15)01238-6

