# Supplementary material for: Treatment patterns and humanistic burden of malignant pleural mesothelioma in Spain
Source: Clin Transl Oncol. 2024 Jul 6;27(1):213–22. doi: 10.1007/s12094-024-03591-5 (PMC11735478; doi:10.1007/s12094-024-03591-5)
Supplement: Supplementary file 1 — Supplementary file1 (DOCX 361 KB) [file 12094_2024_3591_MOESM1_ESM.docx]

SUPPLEMENTARY MATERIAL

Supplementary Figure 1. Study design. CSC, caregiver self-completion questionnaires; eCRF, electronic case report form; EQ-5D-3L, EuroQol 5 dimensions 3 levels; LCSS-Meso, Lung cancer symptom scale-Mesothelioma; PSC, patient self-completion questionnaire; PROs, patient-reported outcomes; WPAI, Work Productivity and Activity Impairment; ZBI, Zarit Burden Interview.

Supplementary Figure 2. Demographic and clinicopathological characteristics of patients with MPM in Spain vs abroad.

Supplementary Figure 3. Reasons for selection of first-line treatment regimen (N=241).

Supplementary Figure 4. Impact of treatment on patients’ daily activities (N=209). Graph shows the frequency with which patients reported their treatment for malignant pleural mesothelioma limited their daily activities. Other treatments were given to 39 patients and are not presented here.

Supplementary Figure 5. Lung Cancer Symptom Scale scores. ASBI, average symptom burden index; HRQoL, health-related quality of life; LCSS, Lung cancer symptom scale. Scores range from 0 (better quality of life) to 100 (worse quality of life).
