# Supplementary material for: Treatment patterns and humanistic burden of malignant pleural mesothelioma in Spain
Source: Clin Transl Oncol. 2024 Jul 6;27(1):213–22. doi: 10.1007/s12094-024-03591-5 (PMC11735478; doi:10.1007/s12094-024-03591-5)
Supplement: Supplementary file 2 — Supplementary file2 (DOCX 26 KB) [file 12094_2024_3591_MOESM2_ESM.docx]

SUPPLEMENTARY MATERIAL

**Supplementary Table 1. Tumour biomarkers detected at baseline.**

| **Tumour biomarker** | **Overall**  n (%) | **Epithelioid histology**  n (%) | **Non-epithelioid histology**  n (%) |
| --- | --- | --- | --- |
| BAP1 | n=4 | n=2 | n=2 |
| BAP1 positive/mutated | 2 (50) | 1 (50) | 1 (50) |
| PD-L1 | n=63 | n=40 | n=23 |
| PD-L1 positive/mutated | 18 (29) | 10 (25) | 8 (35) |
| VEGF | n=14 | n=13 | n=1 |
| VEGF positive/mutated | 1 (7) | 1 (8) | 0 (0) |
| ALK | n=44 | n=31 | n=13 |
| ALK positive/mutated | 2 (5) | 1 (3) | 1 (8) |
| EGFR | n=51 | n=33 | N=18 |
| EGFR positive/mutated | 1 (2) | 0 (0) | 1 (6) |
| ROS1 | n=34 | n=23 | n=11 |
| ROS1 positive/mutated | 0 (0) | 0 (0) | 0 (0) |
| c-MET | n=26 | n=16 | n=10 |
| c-MET positive/mutated | 0 (0) | 0 (0) | 0 (0) |

**Supplementary Table 2. Side effects experienced by patients at the time of data abstraction.**

| **Parameter** | **Overall**  **(N=241)**  **n (%)** |
| --- | --- |
| **Patient currently experiencing side effects from treatment** |  |
| No | 156 (65) |
| Yes | 85 (35) |
| **Anaemia** | 58 (68) |
| **Fatigue** | 50 (59) |
| **Nausea** | 45 (53) |
| **Loss of appetite** | 45 (53) |
| **Weight loss** | 25 (29) |
| **Thrombocytopenia** | 24 (28) |
| **Neutropenia** | 22 (26) |
| **Constipation** | 20 (24) |
| **Vomiting** | 16 (19) |
| **Leukopenia** | 16 (19) |
| **Inability to taste food** | 12 (14) |
| **Hair loss / thinning** | 11 (13) |
| **Mucositis / mouth sores** | 9 (11) |
| **Depression** | 9 (11) |
| **Dry skin** | 8 (9) |
| **Dry mouth** | 7 (8) |
| **Diarrhoea** | 5 (6) |
| **Neuropathy** | 5 (6) |
| **Fever / flu-like symptoms** | 5 (6) |
| **Other muscle pain** | 4 (5) |
| **Rash** | 3 (4) |
| **Other joint pain** | 3 (4) |
| **New / increased trouble breathing** | 3 (4) |
| **Fluid retention** | 2 (2) |
| **Stomach / abdominal pain** | 1 (1) |
| **Headache** | 1 (1) |
| **Mood changes** | 1 (1) |
| **Infections** | 1 (1) |
